# Arsenic in Beer

**Published:** 1901-02-23

**Authors:** 


### ARSENIC IN BEER.

THE adjourned discussion at the Royal Medical and Chirurgical Society on arsenical beer poisoning did not advance matters very much. The symptoms were again described and the proof of the presence of arsenic was confirmed by various speakers, and a good deal of the time of the meeting was occupied in a reiteration of facts which are by this time pretty well known and pretty generally accepted. The conundrum still remains unsolved, however, for Sir Dyce Duckworth still maintained that in his experience the therapeutic use of arsenic was practically free from serious consequences such as were described in the cases before the meeting, even when it was given in large doses.

